# Performance of a Ship-Based Cupronickel Alloy in Exposure Conditions of Arabian Seawater—A Comparative Study

**DOI:** 10.3390/ma17163940

**Published:** 2024-08-08

**Authors:** Syed Ali Sarfraz, Muntazir Abbas, Nasir Mahmood Ahmad

**Affiliations:** 1 Pakistan Navy Engineering College (PNEC), National University of Sciences & Technology, Karachi 75350, Pakistan; sasarfraz@pnec.nust.edu.pk; 2 School of Water, Energy and Environment, Cranfield University, Bedford MK43 0AL, UK; 3 School of Chemical and Materials Engineering (SCME), National University of Sciences & Technology, Islamabad 44000, Pakistan; nasir.ahmad@scme.nust.edu.pk

**Keywords:** polluted seawater, cupronickel 90/10, analytical tools, Raman technique, Arabian Sea, dimensional metrology

## Abstract

Cupronickel-based alloys are widely known for their excellent resistance against aqueous corrosion, however, they can be susceptible to corrosion at accelerated rates and premature failure when exposed to a polluted or brackish seawater medium, even for short-term exposure durations. This unfamiliar corrosion behavior may be a result of the formation of an unprotected corrosion film during the early exposure durations. The paper investigates the corrosion phenomenon in cupronickel 90/10 alloy, by exposing the coupons in two different seawater compositions in the Arabian Sea region. Corrosion losses were investigated on the experimental coupons in a submerged position, for a maximum exposure duration of 150 days, using the conventional weight loss method and a new dimensional metrology-based measurement technique. Additionally, in this research the tubes of a marine heat exchanger having similar material that failed prematurely during operation in the Arabian Sea were also investigated for corrosion losses, followed by the characterization of the corrosion deposits using following analytical techniques: SEM, EDS, XRD and Raman Scattering. The experimental results showed significantly higher corrosion losses on coupons exposed to seawater site rich in pollutants and nutrients including dissolved inorganic nitrogenous compounds, compared to those subjected to a natural seawater solution in corrosion tanks maintained in a controlled environment.

## 1. Introduction

In marine and shipping structures, cupronickel (Cu-Ni) 90/10 alloy is used extensively for seawater pipelines, tubes in seawater-cooled heat exchangers, ships’ cladding, desalination plants and other naval applications because of its excellent heat transfer properties and resistance to biofouling [1,2,3,4,5,6]. The corrosion behavior of Cu-Ni alloy also depends on the seawater chemistry, the levels of aggressive chemical compounds in seawater, nutrients, microbial factors, and pollutants [4,7,8]. Moreover, the intrinsic performance of this material is highly dependent on the presence of detrimental qualities in the seawater, including dissolved inorganic nitrogenous (DIN) compounds, heavy metals, dissolved/undissolved deposits and the water flow rate [8,9,10,11,12,13]. The reviewed literature states that the coolant flow, mixed with pollutants such as plastics, debris and abrasive silicon particles, may rapidly degrade the protective passivating films on the surfaces of Cu-Ni alloys and accelerate erosion–corrosion [14,15].

According to the reviewed literature, the effect of the seawater temperature on the corrosion mechanism in Cu-Ni alloys is not significant [8,10]. Contrary to steels, a rise in the seawater temperature above 27–30 °C may decrease the corrosion in Cu-Ni alloys, mainly due to the formation of a chemically stable oxide layer (copper oxide and/or nickel oxide) at relatively higher seawater temperatures [16,17,18]. These better corrosion protection properties in natural seawater exposure conditions have been attributed to the formation of copper–nickel and iron-based oxide layers [17]. On the other hand, at low seawater temperatures, the formation of protective oxide layers begins at a slow pace, which may require a time period of up to 2–3 months [4,18].

Apart from the detrimental effect of natural climatic factors, corrosion in cupronickel alloys can be accelerated significantly by the addition of pollutants, DINs and sulfur-based compounds [4,8,19,20]. Typically, these aggressive compounds may be added through waste addition directly in harbor/coastal seawater, which may lead to rapid corrosion in Cu-Ni alloy marine structures [21,22,23]

**Figure 1:**
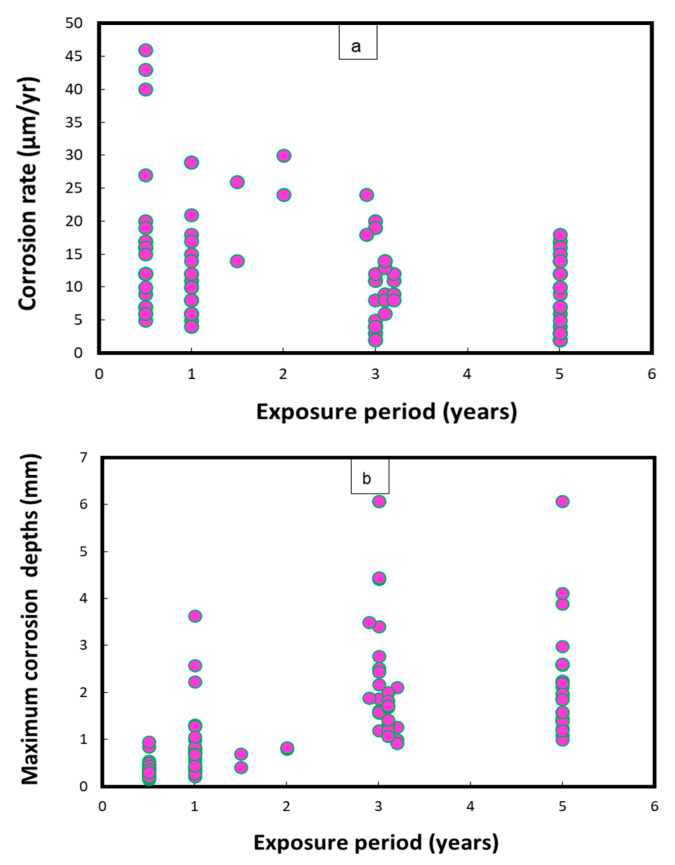
Corrosion parameters recorded for Cu-Ni 90/10 alloy coupons during a worldwide seawater corrosion study [24]. (**a**) Mean corrosion rates; (**b**) maximum corrosion depths.

Figure 1 represents the mean corrosion rates and maximum corrosion depths/pits on uncoated Cu-Ni 90/10 coupons observed during field experiments across 14 global seawater sites [24]. In some cases, the mean corrosion rate may decrease with an increase in the exposure duration, after high early corrosion rates, whereas the corrosion depths (in mm) typically increase proportionally to the duration of exposure. In Figure 1, the mean corrosion rates mostly reach up to 45 µm/y, with the exception of one case in which the corrosion rate was recorded at up to 374 µm/y. In some exposure conditions, significantly higher corrosion depths (1.8–2.6 mm) were observed after exposure periods between 3 and 5 years, mostly in the harbor seawaters of Port Hueneme, CA (USA), and Innisfail, Queensland (Australia). Moreover, the highest corrosion rates and maximum corrosion depths for carbon steel coupons were observed in the seawaters of Port Hueneme, California. Some water quality reports have indicated considerable nitrate levels (up to 10 ppm) and low DO concentrations in the seawater conditions of Port Hueneme, possibly due to the presence of various pollutant species [25,26,27]

Some researchers have identified that the outermost corrosion layers develop from the precipitation of dissolved copper species, whereas the innermost layers are mainly composed of cuprite (Cu_2_O) [28].

The reddish-colored corrosion product that appears in the Cu-Ni alloys typically corresponds to cuprite, whereas the turquoise blue color corresponds to basic copper chlorides (Cu_2_(OH)_3_Cl) that develop on top of the cuprite layers [29]. Tenorite (CuO) is another copper oxide that normally forms when Cu-Ni alloys are exposed to seawater. It is a comparatively unstable compound that forms as an early corrosion product on copper and Cu-Ni alloys, and this then transforms rapidly into more stable compounds with further exposure [29,30]. These copper chlorides exist in several polymorphs, such as clinoatacamite, atacamite, paratacamite, botallactite and nantokite. In Cu_2_(OH)_3_Cl, the crystalline structures of different polymorphous compounds vary, e.g., botallackite is monoclinic prismatic and clinoatacamite is pseudo-rhombohedral, whereas atacamite shows an orthorhombic structure [29,31].

In marine environments, the proportion of cuprite layers on the metal surface declines with time, followed by an increase in the basic chloride content in the compounds. Cuprite typically forms as a nanometric thin film at the early stages via the direct oxidation process. It then reacts with the chlorides and this may lead to the formation of a cuprous chloride, e.g., nantokite (CuCl), which further transforms into clinoatacamite (Cu_2_(OH)_3_Cl) as an end corrosion product [32]. Clinoatacamite is considered as the most stable polymorph, whereas other phases are transitional in a series of reactions, and it forms clinoatacamite as the end product [29,30,31]. The cupric/cuprous ions may form stable complex compounds (polymorphs) in the presence of high chloride levels (i.e., Cl^−^); therefore, the concentration of Cu ions may be reduced significantly [29,33]. Some researchers have predicted the typical stratification of the corrosion product layer (as a function of the DO concentration) composed of cuprous chloride (CuCl), cuprous oxide, cupric hydroxide/oxide and atacamite/malachite from the innermost to outermost layers [34]. The passive layer formation on Cu-Ni alloys in natural seawater may take place in sequential structures of Cu_2_O, CuO and Cu (OH), depending upon the depth of the corrosion layer [29,35].

This research aims to investigate the corrosion trends and differences in the corrosion loss behavior of Cu-Ni alloy 90/10, in the form of round coupons, by exposing them to natural and pollutant-rich seawater sites in the Arabian Sea off the coast of Karachi. This research also investigates the corrosion losses on the tubes of a ship-based marine heat exchanger (Cu-Ni 90/10 alloy) that failed after operating for a duration of up to 10 years in harbor seawater rich in pollutants including nutrients and DINs. Furthermore, the corrosion deposits on post-corrosion coupons and tubes are characterized for their elemental compositions and compounds using various analytical tools, such as SEM, EDS, XRD and Raman scattering techniques.

## 2. Experiment and Methods

**Figure 2:**
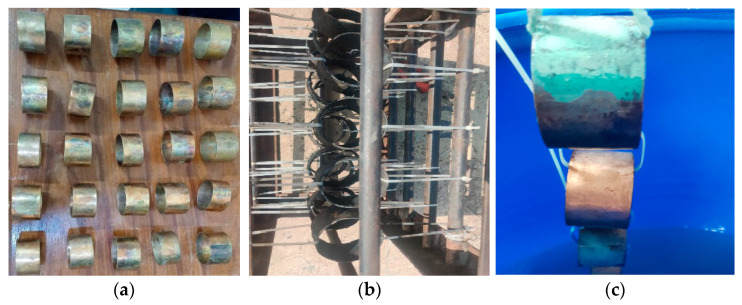
(**a**) Surfaces of prepared pre-corrosion experimental coupons of Cu-Ni 90/10 alloy; (**b**) coupon placement in harbor seawater site; (**c**) coupon placement in corrosion tank containing natural seawater from the Arabian Sea.

The experimental coupons of Cu-Ni 90/10 were prepared in circular rings by cutting from long pipes (having a thickness of 1.6 mm and inner radius of 31.6 mm). After surface finishing (sand papers of up to 400 U.K. grits), cleaning and weighing, these coupons were then exposed to (i) a polluted seawater site in a harbor and (ii) the clean natural seawater of the Arabian Sea contained in a corrosion tank, in a controlled laboratory environment onshore, situated quite close to the coast. It was assumed that the environmental factors maintained in the corrosion tank were similar to those in the field at the natural seawater site. The seawater chemistry in the latter experimental site was maintained and the seawater was replaced whenever the water chemistry/quality began to degrade. Coupons dipped in the polluted seawater were placed in a rectangular box connected with the help of nonmetallic cable ties, as shown in Figure 2.

Table 1 shows the material compositions for the experimental coupons and marine heat exchanger tubes evaluated using the energy-dispersive spectroscopy (EDS) and atomic (spark) emission spectroscopy (AES) methods.

Here, the average corrosion rates, corrosion losses and maximum corrosion pit depths were measured using dimensional metrology (DM), as well as the typical standard weight loss methods [36,37,38]. A standard formula in line with ASTM standard G1-03 from Ref. [38] was used for the average corrosion rate measurement. The surfaces of the experimental coupons before and after seawater exposure were cleaned as per ASTM standard G52 [39].

The coupons were placed in a submerged position in both seawater sites throughout the experiment. The assumptions made during experimentation included a similar seawater temperature variation during the corrosion tests at both sites and the similarity of the seawater quality at each site. In the case of the marine heat exchanger tubes, localized thickness losses were measured with the help of the DM method [37]. Here, the localized corrosion depths were calculated on the seawater side with a 1.2 mm thickness, which was perforated after ten years of service life, mostly in the polluted harbor seawater in the Arabian Sea off the coast of Karachi.

Table 2 shows the specifications of the seawater samples taken from both experimental setups.

Weight measurements on pre-exposed coupons were taken using an analytical weighing scale (accuracy of up to four digits after decimal). The post-exposure experimental coupons were collected after every 15 days for the first two months, and thereafter on a monthly basis, for a maximum of 150 days. Prior to the weight loss calculations, the post-corrosion coupons were cleaned according to the ASTM standard [38].

**Figure 3:**
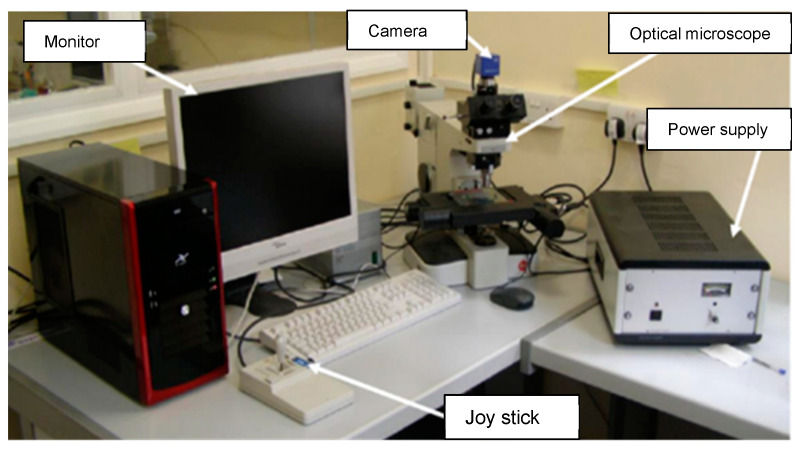
Setup for DM–image analyzer integrated with optical microscope.

**Figure 4:**
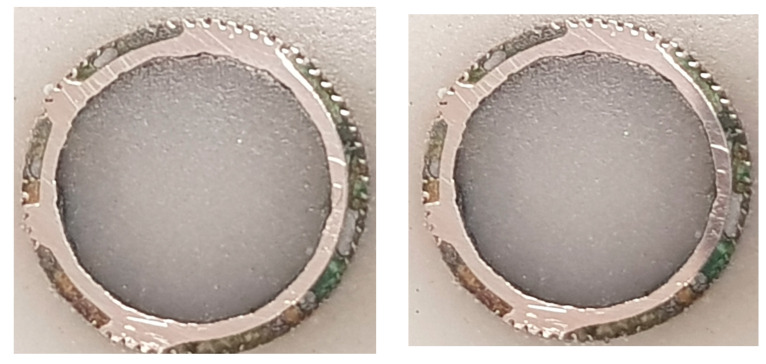
Prepared cross-sections of the tubes of a failed marine heat exchanger exposed mainly in the polluted seawater site in the Arabian Sea.

For the heat exchanger tubes, which failed during operation in a ship refrigeration system after 10 years of service life, the cross-sections were prepared from the tubes by cutting them into circular rings, for the evaluation of the corrosion depths, using a specialized optical microscopy/image analyzer arrangement integrated with the DM technique, i.e., the DM–image analyzer approach, as shown in Figure 3. The cross-sections prepared from the heat exchanger tubes and evaluated for thickness loss measurement are shown in Figure 4.

The DM–image analyzer method was used for the thickness loss measurements in the corroded coupons and heat exchanger tubes. The thickness loss data are represented as a cumulative probability (in terms of percentages and standard deviations). The cumulative probability is determined using the following equation.

(1) $$\mathrm{Cumulative}\;\mathrm{probability}\;(\%)=\frac{n}{N+1}\times 100$$

Here, ‘*n*’ represent the rank number and ‘*N*’ is the total number of thickness loss points measured on each coupon.

The corrosion damage morphologies and elemental characterization of the corrosion products were carried out with the help of a TESCAN VEGA3 microscope (at 10–20 Kv). SEM images of the various corrosion phases were acquired at magnification of up to 20,000×. The unique inbuilt electron optics in VEGA3 include an intermediate lens (IML) to enable frequent switching from low to high magnification and low to high voltages within a few seconds. These features make sample imaging faster and more reliable. This SEM also provides a larger chamber to hold 6 samples, each with a maximum sample size of 1 inch.

In this system, the EDS technique helps to investigate the elemental compositions of the base metal and deposited corrosion product and allows for EDS mapping. It facilitates both qualitative and quantitative estimations of the elements and metal oxides in different corrosion products and deposit layers.

For the evaluation of the elemental composition using EDS mapping, the corroded coupons of the heat exchanger tubes were mounted in a resin mixture without removing the deposited corrosion layers. For X-ray diffraction, a Siemens X-ray diffractometer (D5005) was used, and data were collected over a 2θ range between 10° and 90°. The duration of each run was about 1 h. In this research study, Raman spectroscopy was performed at room temperature using a LabRAM Horiba spectroscope, with monochromatic (red) laser light (632 mm He/Ne) in the wavelength shift range of 100–1100 cm^−1^.

## 3. Experimental Results and Discussion

**Figure 5:**
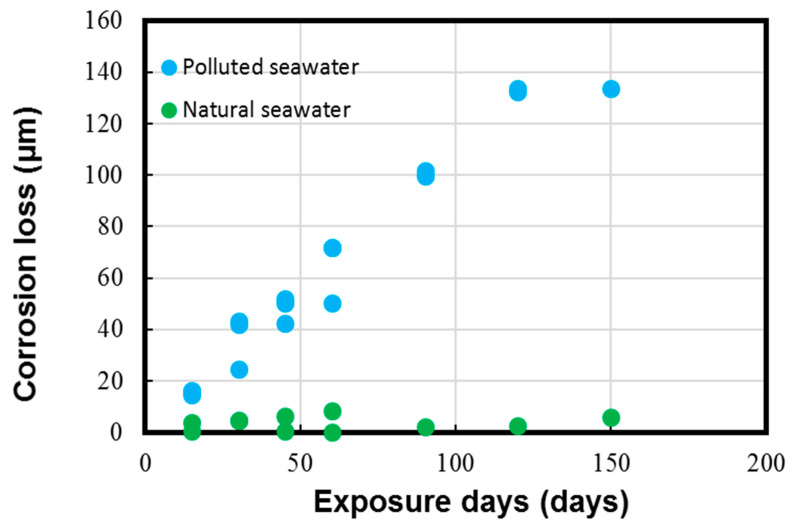
Corrosion loss parameter measured for Cu-Ni 90/10 alloy coupons. Reprinted with permission from [40].

**Figure 6:**
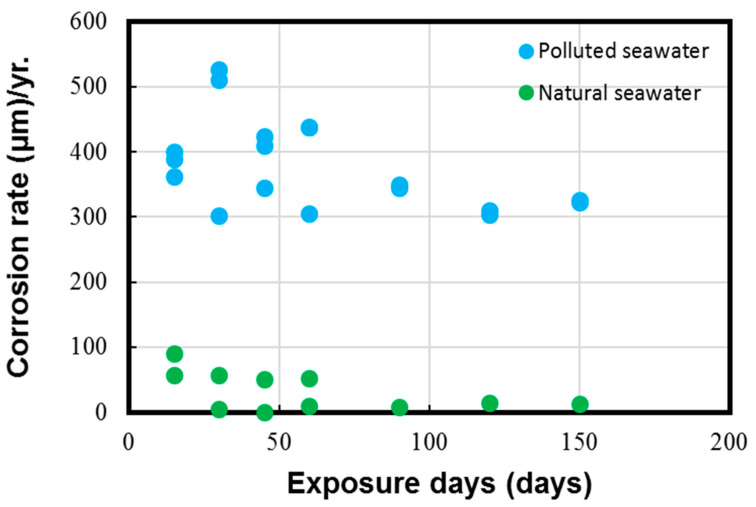
Corrosion rate parameter measured for Cu-Ni 90/10 alloy coupons. Reprinted with permission from [40].

The average corrosion loss and corrosion rate data calculated on the experimental coupons with the help of the typical weight loss method are shown in Figure 5 and Figure 6, respectively.

The results in Figure 5 and Figure 6 depict the corrosion rate parameter (ranging from 300 to 550 µm/y), which is significantly higher in the polluted seawater site and is rich in DINs and other influential corrosion factors, such as low pH levels and low DO content (as shown in Table 2). Likewise, the corrosion losses noted in the natural seawater site were quite nominal (ranging from 10 to 60 µm/y). These measured parameters in the natural seawater were found to be similar to those previously reported in similar seawater sites, as reported in Figure 1 in Phull et al. [24], as well in other reviewed literature [8,13,41]. On the other hand, the corrosion losses in the polluted seawater site were extremely high and comparable to those reported previously in similar seawater compositions predominantly rich in pollutants [1,7,8,24].

**Figure 7:**
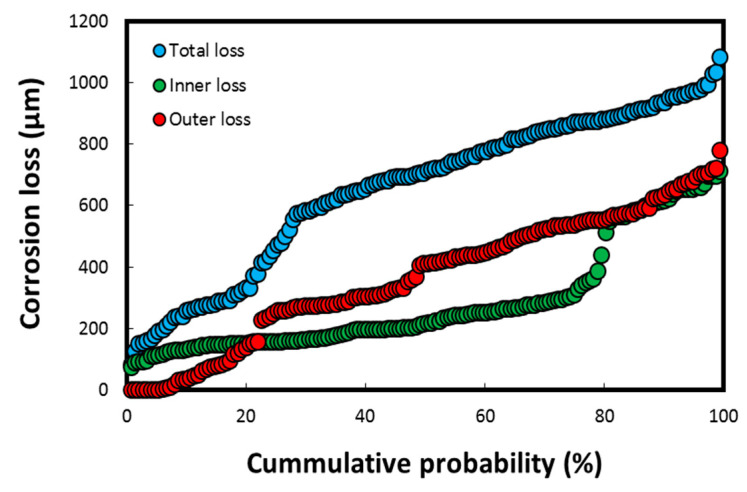
Cumulative probability (%) parameter for corrosion loss on heat exchanger tubes calculated using DM–image analyzer technique, both on seawater side (inside tube) and gas side (outside tube) of heat exchanger. Reprinted with permission from [40].

The cumulative probability (%) of the thickness losses/depths on failed heat exchanger tubes (having a tube thickness of up to 1.2 mm), measured using the DM–image analyzer technique, is shown in Figure 7.

The thickness losses in Figure 7 were observed mostly on the tubes exposed to the polluted harbor seawater, which subsequently led to the perforation/failure of five tubes (total tube thickness of 1.2 mm). These values are significantly higher for a corrosion-resistant material such as Cu-Ni 90/10, in which the mean corrosion loss is reported to be well below 50 µm/y (the typical range in clean natural seawater is 2–12 µm/y at a flow velocity below 3 m/s) in natural seawater exposure conditions [4,8,24,42]. The cumulative corrosion depth data indicated in Figure 7 corresponds to the pitting data resulted in the loss of integrity of the heat exchanger tubes. Hence, these results imply that the overall corrosion rate on the seawater side of the tubes is approximately 100 µm/y, which is again considerably higher for a corrosion-resistant material such as Cu-Ni 90/10 exposed to a natural seawater environment. However, it is not unusual in a harbor seawater environment flooded with untreated waste water and pollutants from multiple sources, including heavily polluted sewage effluents [7,8,15,24].

**Figure 8:**
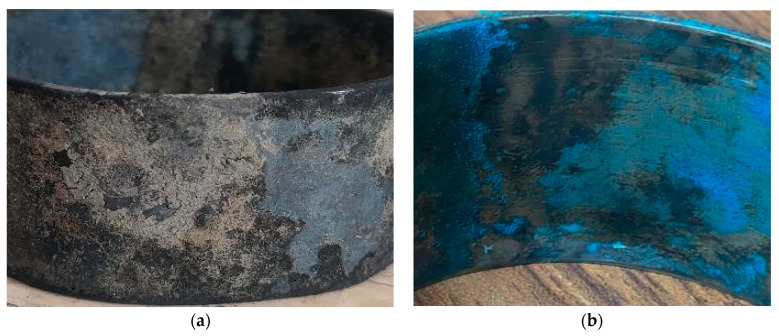
Macroscopic view of corroded coupons of Cu-Ni 90/10 material collected from (**a**) polluted seawater site and (**b**) natural seawater site.

**Figure 9:**
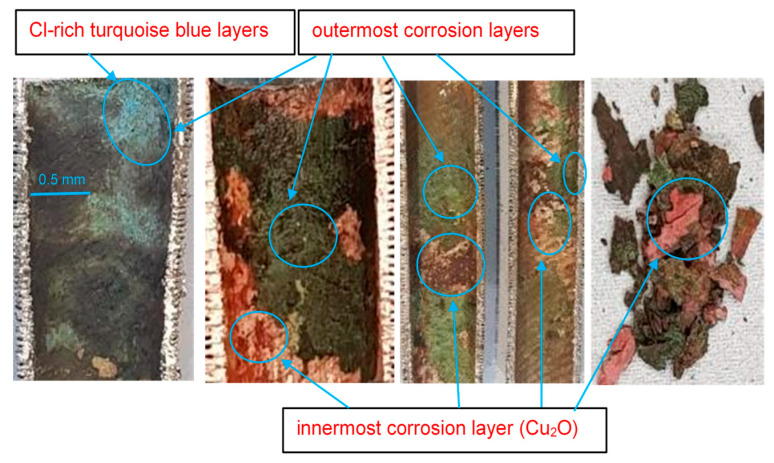
Macroscopic surface morphologies of corrosion layers on Cu-Ni 90/10 alloy tubes operating in seawater conditions of Indian Ocean.

Figure 8 and Figure 9 show the post-corrosion coupons and tubes recovered from both the polluted and natural seawater sites. A blackish corrosion layer was deposited on the coupons placed in the harbor seawater site, whereas bluish deposits appeared on the coupons recovered from the natural seawater-containing tank after 150 days.

**Figure 10:**
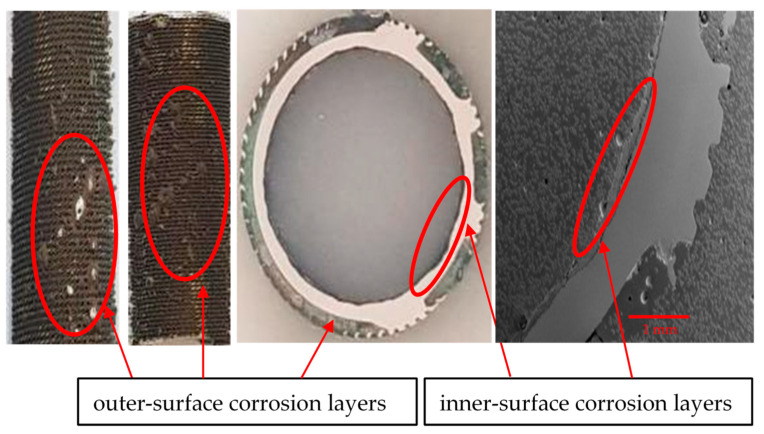
Inner and outer surfaces of heat exchanger tubes with corrosion deposit layers and thickness losses.

In Figure 9, the outermost corrosion layers (away from the tubes’ metal surfaces) present blackish, greenish and turquoise blue colors. The turquoise blue appearance of the external corrosion surface typically indicates the presence of high chloride content [29]. Underneath the top corrosion layers, a highly intact reddish corrosion layer is observed near the alloy surface. This reddish corrosion layer (Figure 9) represents a cuprite film, which normally develops in low-salinity regions. Figure 10 presents the macroscopic images of the external surfaces of the extracted tubes; these are normally exposed to the less corrosive refrigerant gas (R-22 or R-34). It is, however, evident from the photographs that the outer finned surfaces of the tubes are also severely affected by the corrosion. The greenish rust layers (cupric/cuprous oxides) and localized corrosion marks are clearly visible on the exterior surfaces of the tubes, probably indicating the localized corrosion depths or leaked/perforated regions.

**Figure 11:**
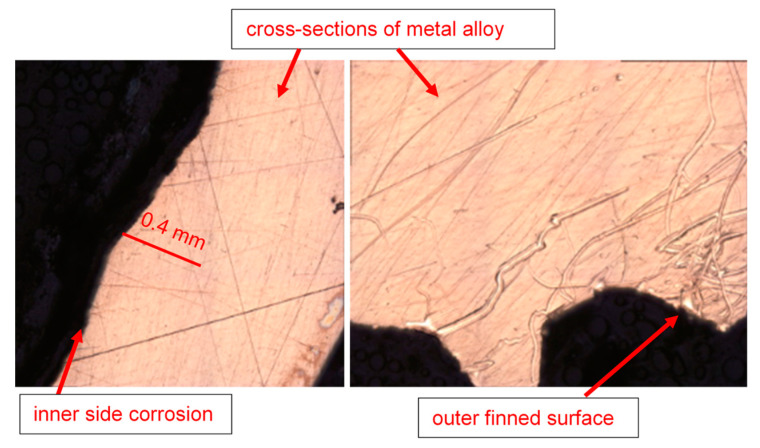
Inner and outer topologies of a corroded tube cross-section viewed under an optical microscope (using 20X lens). The gear-shaped metallic structures are the finned structures on the outer surfaces of the tube.

The optical microscopic images of the tube cross-sections (inner and outer sides) are shown in Figure 11. From several corroded tubes, only five cross-sections were prepared for inspection, as random samples out of the total population of the failed tubes. After the failure of the tubes, it was assessed (based on engineering experience) that the maximum metal loss and subsequent tube perforation occurred mainly due to seawater corrosion, which affected the tubes at a pressure of around 30–35 psi.

**Figure 12:**
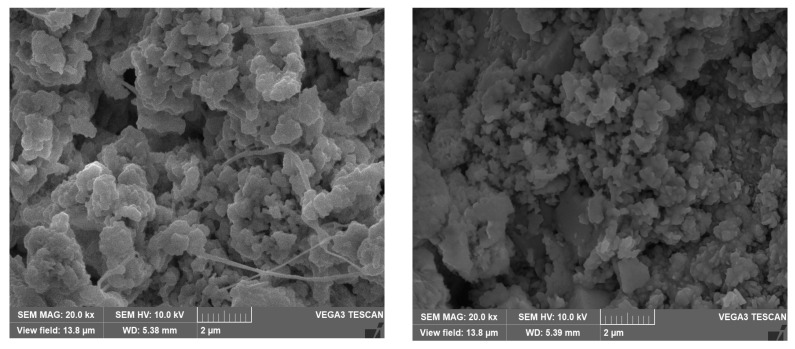
SEM micrographs of the outermost corrosion products/deposits on the heat exchanger tubes at magnification of 20k.

**Figure 13:**
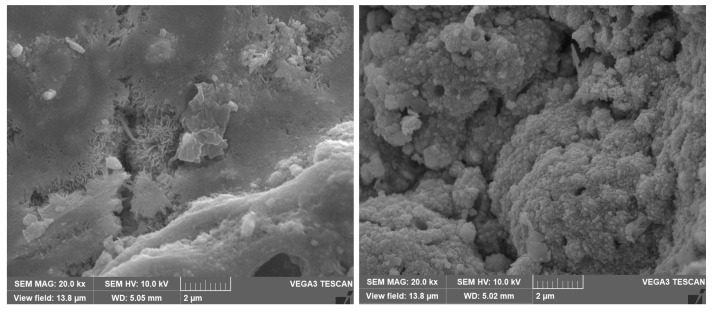
SEM micrographs of the corrosion deposits (on Cu-Ni 90/10 alloy tubes) on the innermost corrosion layers (reddish layers), at magnification of 10 kx and 20 kx.

The SEM micrographs of the corrosion products/deposits collected from the seawater side (inner side) of the tubes are shown in Figure 12 and Figure 13. In some cases, different crystalline structures developed within the cracked corrosion surfaces and pores.

**Figure 14:**
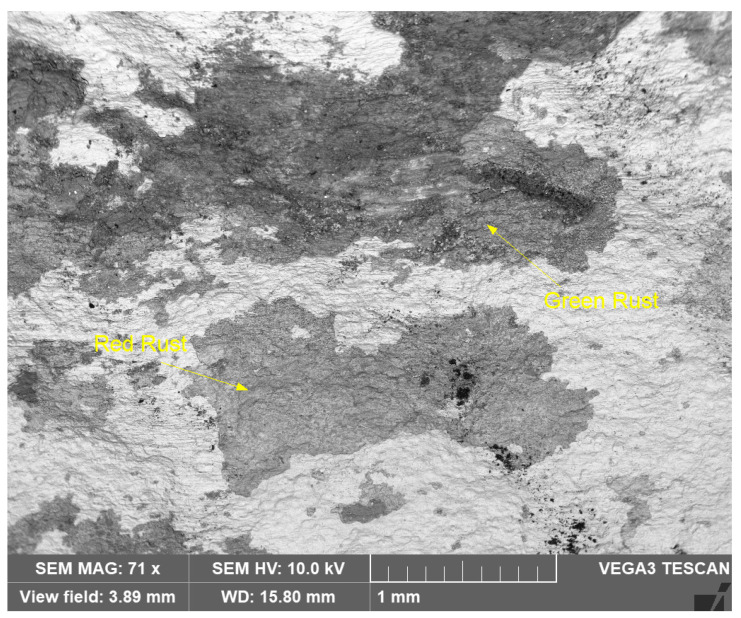
SEM micrograph of greenish and reddish corrosion patches (shown in Figure 9) on seawater side of Cu-Ni 90/10 alloy tube (BSE view).

Similarly, Figure 14 shows an SEM image of the reddish and greenish corrosion patches (shown in Figure 9), at lower magnification (71X).

**Figure 15:**
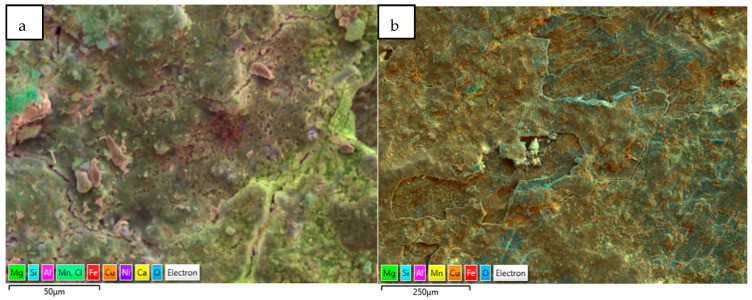
(**a**,**b**) Elemental map (EDS mapping) of the corrosion products (next to metal surfaces) collected from marine heat exchanger tubes.

Another form of elemental distribution in the corrosion products on the seawater side of the tube (inner) is shown in Figure 15, obtained using SEM-EDS layered images and analyses. In this technique, the most significant (wt. %) elements in the corrosion product have been highlighted with different colour shades, without changing their actual positions, so that the positioning of various elements in the corrosion product can be evaluated with respect to the metallic surfaces.

**Figure 16:**
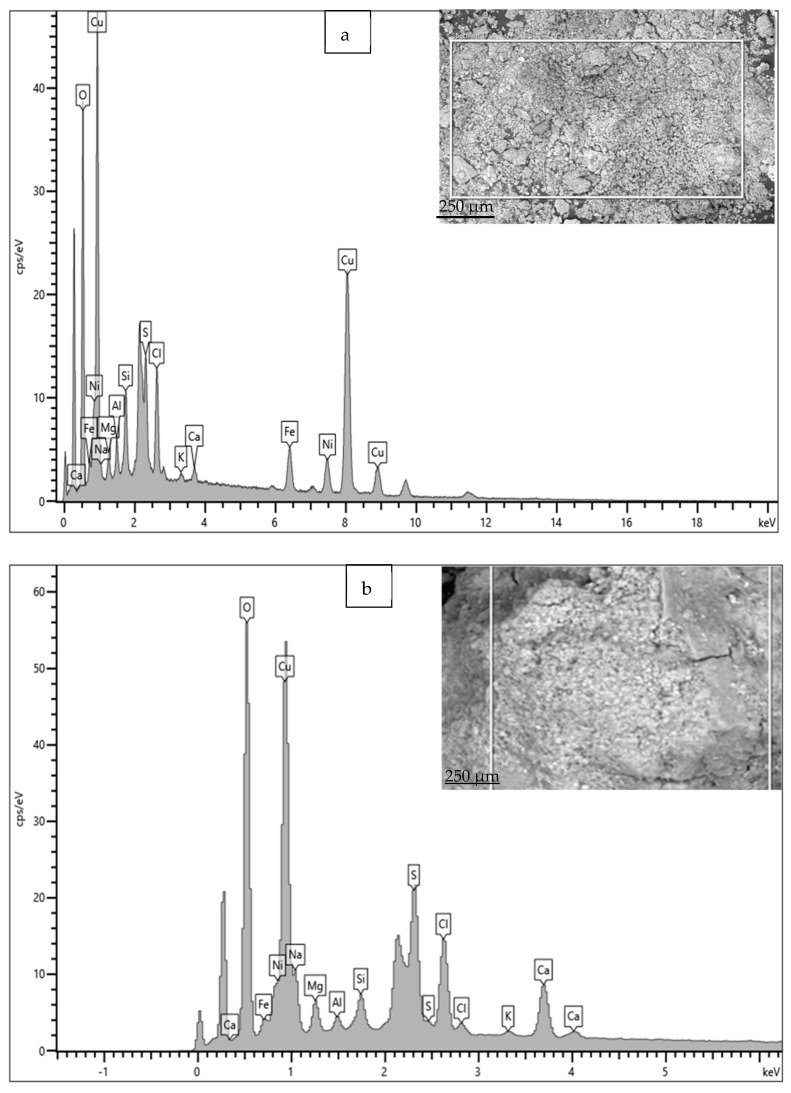
(**a**,**b**) EDS results for corrosion products deposited on heat exchanger tubes exposed mainly to the polluted seawater conditions of the Arabian Sea.

The EDS spectrum and elemental composition details are given in Figure 16 and Table 3. Apart from the elements in the material composition, the significant presence of S, Cl, Ca, Si and Mg in the rust is also observed. In particular, the presence of S and Cl is significantly high; these are widely recognized as causing rapid corrosion (localized) losses in Cu-Ni alloys, because S and Cl may result in the formation of various corrosive compounds (sulfides, complex chlorides, etc.), which have detrimental effects on the corrosion resistance of Cu-Ni alloy structures [2,14]. The presence of Fe in the corrosion products is significant (up to 4.5–5.8 wt. %), despite its low presence in the material composition (up to 1 wt. %).

**Figure 17:**
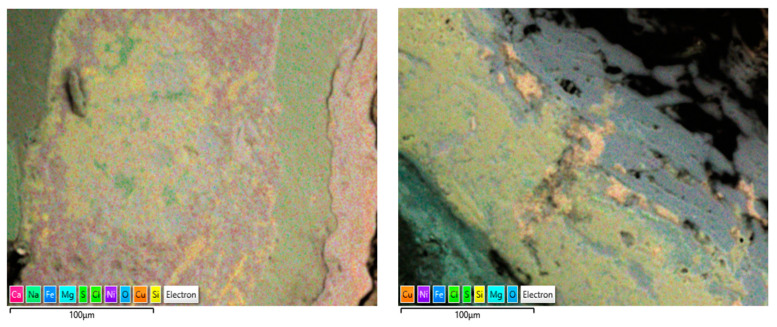
EDS elemental maps of the corrosion deposits accumulated on the seawater side of the heat exchanger tubes exposed mainly to the polluted seawater site (both images show corrosion on tubes exposed to the same polluted seawater sites).

The corrosion products on the failed tubes of Cu-Ni 90/10 were also examined using EDS mapping. In addition to obtaining the EDS spectra for the elemental compositions (wt. %), EDS mapping also enables the visualization of the positions of the accumulated elements in the corrosion products, which may reflect the exposure conditions (i.e., seawater constituents). Figure 17 shows some of the EDS maps illustrating the distribution of the elements in the corrosion product accumulated on the seawater side of the condenser tubes (Cu-Ni 90/10) operated in the polluted seawater region in the Arabian Sea. As well as other elements, the substantial presence of sulfur and chlorides is visible in both images; these were also visible in the typical EDS (wt. %) results.

**Figure 18:**
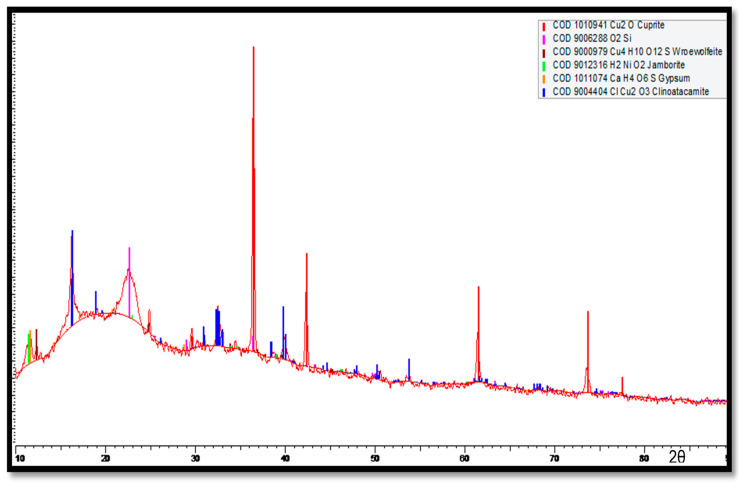
XRD analysis of the multi-colored corrosion products deposited at the outermost corrosion layers on heat exchanger tubes exposed to the polluted seawater site in the Arabian Sea.

The XRD results for the corrosion products accumulated on the Cu-Ni 90/10 tubes at the outermost layers (multi-colored) are shown in Figure 18. Here, major peaks are identified for the cuprite, clinoatacamite and SiO_2_ compounds. Lower levels of jamborite nickel oxide/hydroxide, gypsum and paratacamite are also observed.

**Figure 19:**
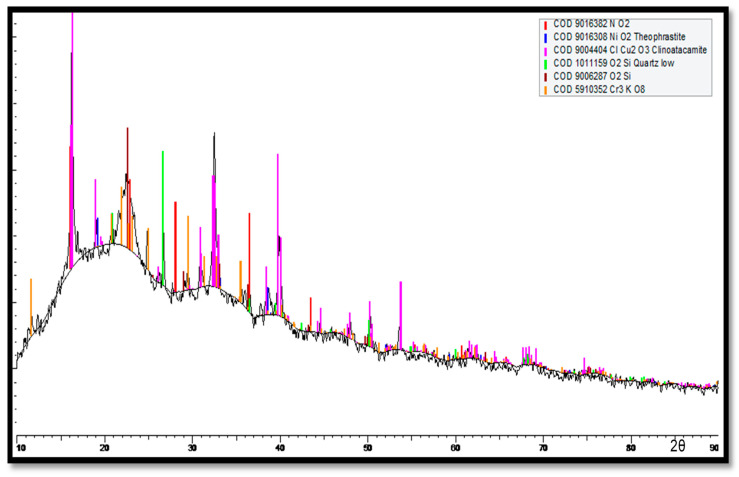
XRD analysis of corrosion products (reddish products next to metal surfaces) deposited next to the surfaces of Cu-Ni 90/10 alloy tubes.

For the innermost corrosion layers (reddish) observed on the tubes exposed to the polluted seawater of the Arabian Sea, the XRD spectrum is shown in Figure 19. Clinoatacamite and quartz (SiO_2_) appeared as major compounds. Lower levels of paratacamite, aluminum, cuprite, tenorite and theophrastite (NiO_2_) were also observed.

In Figure 19, high cuprite content can be observed on the outermost corrosion layers (seawater sides of the tube), whereas clinoatacamite was found in abundance in the innermost corrosion layers. In the presence of high salinity (Cl^−^ ions) exposure, the cuprite layers transform into various polymorphs (e.g., botallackite and atacamite) of copper chlorides, which are subsequently transformed into clinoatacamite, being the most stable phase.

**Figure 20:**
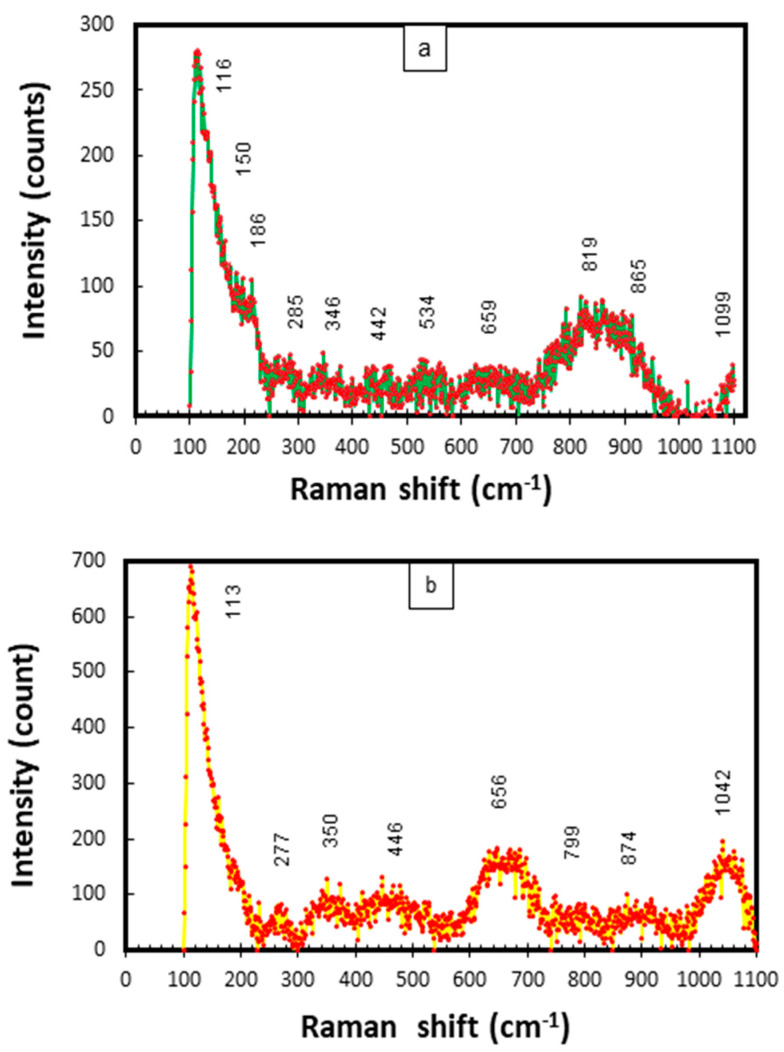
(**a,b**) Raman scattering plots for corrosion products collected from CuNi−90/10 alloy tubes exposed to polluted seawater conditions in the Arabian Sea.

In this study, the frequencies of all Raman scattering bands observed (for Cu-Ni 90/10) were compared to the reference values given in the published literature for the probable corrosion compounds on Cu-Ni alloys exposed to marine conditions [34,43,44,45,46]. The combined Raman scatter plots for mixed corrosion products/deposits (on the inner and outer layers on the seawater side) are given in Figure 20.

In Figure 20a,b strong characteristic bands of Cu_2_O and NiO can be observed at 150, 446 and 526 cm^−1^. The peaks at 534, 1042 and 1099 cm−1 may also represent NiO. The bands at 113–116 cm^−1^ may be attributed to aragonite. The peaks at 278, 285, 344 cm^−1^ and 351 cm^−1^ have also been linked to CuO [46]. Moreover, the peaks at 819, 865, 874, 1044 and 1099 cm^−1^ can be attributed to Cu(OH)_2_, Cu_2_(OH)_3_Cl and CuCl/CuCl_2_, respectively. In the published literature, strong peaks at 890, 942 and up to 1100 cm^−1^ have been attributed to the clinoatacamite and paratacamite compounds, respectively [31]. The presence of both of the above compounds has also been verified by the XRD results. A strong peak in the band of 346–350 cm^−1^ may be attributed to iron sulfide (FeS). Peaks at 460 and 527 cm^−1^ have been recognized for jamborite in a previous research study [47].

This research work was conducted for short duration of a maximum of 150 days, but this needs to be increased to a period of up to 1–2 years for a better understanding and the subsequent formulation of corrosion models and the remaining useful life assessment of cupronickel-based marine infrastructure exposed to the Arabian Sea near coastal regions, affected by unregulated marine pollution and various species of detrimental chemical compounds.

## 4. Conclusions

The corrosion test results for the experimental coupons and the failed heat exchanger tubes reveal the following.

- Significantly higher average corrosion rates (ranging between 0.3 and 0.5 mm/y and decreasing with an increase in exposure duration) were observed on coupons exposed to pollutant-rich harbor seawater, whereas the corrosion rates observed in natural seawater conditions were far lower (ranging between 12 and 50 µm/y and decreasing with the exposure duration). The latter were found similar to those reported in the previously published literature for natural seawater conditions.
- The chemical composition of the polluted harbor seawater, primarily due to the higher levels of DINs and sulfur-containing compounds and relatively lower levels of pH and DO, can be the major cause of accelerated corrosion rates and losses.
- After an exposure duration of 150 days, among the experimental coupons, approximately five- to six-times higher average corrosion losses and corrosion rates were observed in the Cu-Ni 90/10 coupons placed in the polluted seawater compared to those in the natural seawater.
- On the basis of the spectroscopic analysis and characterization, these higher corrosion losses may be attributed to the significant role of DINs, sulfur compounds and the chloride content in the polluted harbor seawater site.
- The DM-based corrosion measurement approaches (micrometry and image analyzer) provided localized corrosion loss data of superior quality in the form of a corrosion depth profile, especially in the case of the heat exchanger. The thickness loss data measured using DM-based methods are more realistic and instrumental (than the average mass loss data) in statistical analyses for corrosion prediction modeling and remaining useful life/reliability estimations.
- Spectroscopic techniques provide highly useful data for the characterization of deposits, the elemental composition and the compounds formed on heat exchanger tubes exposed to polluted seawater for a duration of more than ten years. In the EDS graphs and EDS mapping, significantly higher S and Cl levels were observed in the corrosion deposits accumulated on the failed tubes of the heat exchanger exposed to the polluted seawater site.

## Figures and Tables

**Table 1:** Elemental compositions (wt. %) of experimental coupons and heat exchanger tubes of Cu-Ni 90/10 material.

Cu	Ni	Fe	Mn	Al	Si
R	9.95	1.14	0.88	0.13	<0.09

**Table 2:** Seawater specifications at the study sites [36].

Parameters	Natural Seawater	Polluted Seawater
Temperature (°C)	25–30	25–30
pH Level	7.5–8.2	6.9–7.4
DO (mg/L)	>3.5	0.63 ± 0.39
EC (mS/m)	50	63 ± 4.8
Nitrates (mg/L)	<0.1	1.2 ± 0.3
Chloride (mg/L)	18,000 ± 500	21,000 ± 1000
Sulfate (ppm)	1900	2900

**Table 3:** Elemental compositions (wt. %) calculated (using EDS) for the corrosion products/deposits on Cu-Ni 90/10 tubes exposed to polluted seawater conditions.

O	Na	Mg	Al	Si	S	Cl	K	Ca	Fe	Ni	Cu
41.9	3.3	1.8	2.3	4.8	3.3	2	0.6	0.9	5.8	2.9	30.1
40.5	2.9	1.7	0.5	1.0	4.8	3.3	0.2	2.17	2.9	1.8	38.1

## Data Availability

The original contributions presented in the study are included in the article, further inquiries can be directed to the corresponding author.
